# “The 2-Dimensional Approach”: a novel tool to help learners organize their knowledge and improve their clinical reasoning skills.

**DOI:** 10.15694/mep.2019.000134.1

**Published:** 2019-06-17

**Authors:** Hisashi Shimozono, Makoto Takahashi, Makoto Tomita, Kazuki Takada, Yujiro Tanaka

**Affiliations:** 1 Department of Medical Education Research and Development; 2 Clinical Research Center; 3 Department of Professional Development in Health Sciences

**Keywords:** the 2D Approach, novel tool, visualization of pathophysiology, knowledge organization, clinical reasoning, diagnosis.

## Abstract

This article was migrated. The article was marked as recommended.

**Background:** Clinical reasoning is an essential competence of a physician. Particularly, pathophysiological understanding is the key for novices to organize their knowledge and improve clinical reasoning. To this end, we propose a “2-Dimensional (2D) Approach” that visualizes pathophysiology by using a matrix of organs and systems.

**Methods:** This study was a prospective observational study of 100 residents (PGY-1), who attended a lecture on the 2D Approach. They underwent a pre- and post-test that assessed their ability to list differentials for various symptoms and to choose the most likely diagnosis for a case vignette. We stratified the participants according to their baseline knowledge level on the pretest into 2 groups (high-knowledge and low-knowledge). In each of the 2 groups, we compared the change in the pre- and post-test scores between participants who used the 2D Approach during the posttest and those who did not.

**Results:** In the high-knowledge group (n=59), the change in the pre- and post-test scores was significantly larger in the 2D Approach users than non-users (p=0.046), while in the low-knowledge group (n=41), there was no significant difference in the change of the scores between the 2D Approach users and non-users.

**Conclusions:** On condition that residents have sufficient knowledge, the 2D Approach helps them organize their knowledge and improve their clinical reasoning skills, through visualization of pathophysiology on a matrix of organs and systems.

## Introduction

Clinical reasoning is an essential competence of a physician ( Norman, 2005) and it has been investigated for more than 3 decades ( Croskerry, 2009; Norman, 2009). For expertise, organizing knowledge is considered to be an important process. Especially, an illness script is the most encapsulated form of knowledge that is applied to pattern recognition ( Charlin *et al.*, 2007).

On the other hand, clinical reasoning needs to be integrated with basic science knowledge ( Woods, 2007; Woods and Mylopoulos, 2015; Lisk, Agur and Woods, 2016). Particularly, pathophysiological understanding is the key for novices to organize their knowledge and improve their clinical reasoning skills ( Schmidt and Rikers, 2007; Schmidt and Mamede, 2015).

Although some remediation are proposed ( Chamberland *et al.*, 2011; Guerrasio and Aagaard, 2014) for many difficulties in developing clinical reasoning skills ( Audetat *et al.*, 2013), visualizing the reasoning process is necessary but challenging ( Delany and Golding, 2014).

**Figure 1.**
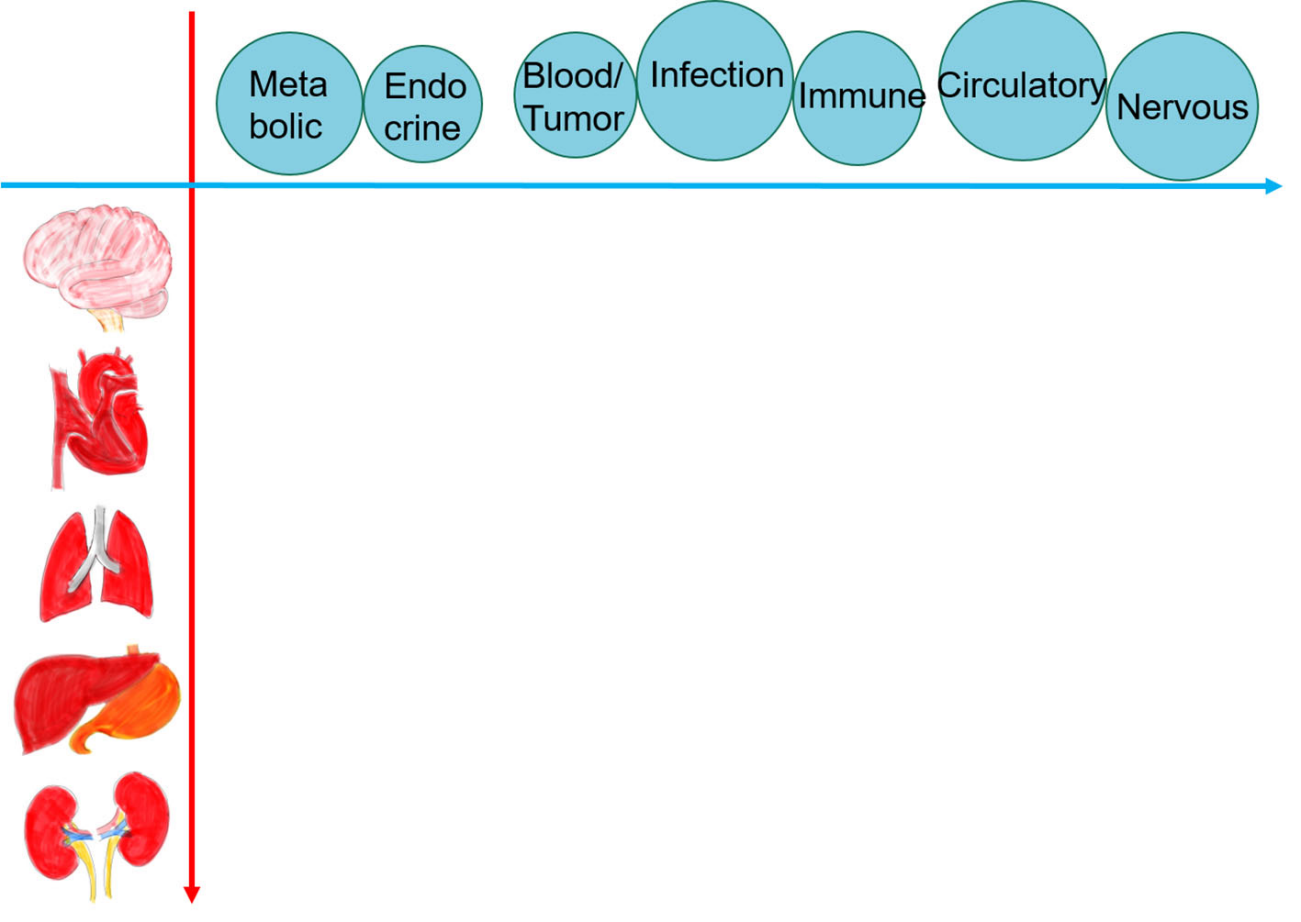
The 2D Approach uses a 2-dimensional matrix with organs and systems.

To this end, we propose a new methodology called “the 2-Dimensional (2D) Approach”, which visualizes pathophysiology by using a matrix of organs and systems. According to Harrison’s Principles of Internal Medicine ( Kasper *et al.*, 2005), internal medicine consists of neurology, cardiology, pulmonology, gastroenterology, nephrology, endocrinology and metabolism, oncology and hematology, infectious diseases, and rheumatology. Physicians need enormous knowledge to make a diagnosis in patients with diverse medical problems. We therefore combine these entities into 2 large categories, organs and systems, and propose a 2-dimensional (2D) matrix using these categories ( Figure 1). We presented this method under the name of “5x5 Approach” in AMEE 2018 ( Shimozono, Takahashi and Tanaka, 2018).

In this approach, the brain (neurology), heart (cardiology), lung (pulmonology), liver/intestine (gastroenterology), and kidney (nephrology) are classified as organs, and the metabolic (metabolism), endocrine (endocrinology), blood/tumor (oncology and hematology), infection (infectious disease), immune (rheumatology), circulatory, and nervous systems are classified as systems. The circulatory system and the heart are included under cardiology, but the circulatory system spreads from the heart and causes many diseases in multiple organs including the heart itself (e.g., myocardial infarction). Similarly, the nervous system and the brain are under neurology, but abnormality in the nervous system causes abnormal symptoms in many organs.

The basic concept of the 2D Approach is that the systems involve throughout the whole body and can be pathogenesis of diseases, while the organs show vital signs and symptoms as a consequence of diseases. This cause-and-effect relationship between the organs and the systems on the 2D matrix allows users to visualize the underlying pathophysiology of a patient and investigate a broad array of pathophysiological diagnostic hypotheses. To explain how the 2D Approach visualizes the pathophysiology and clinical reasoning process using a matrix of organs and systems, we present 2 examples, and compare it with existing methods.

### Visualization of pathophysiology

#### Case 1

**Figure 2.**
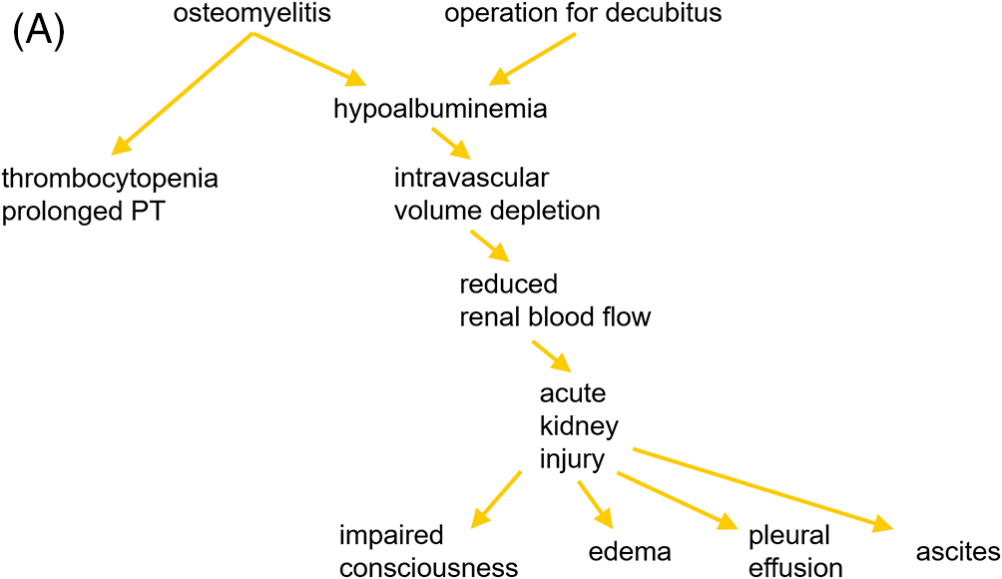

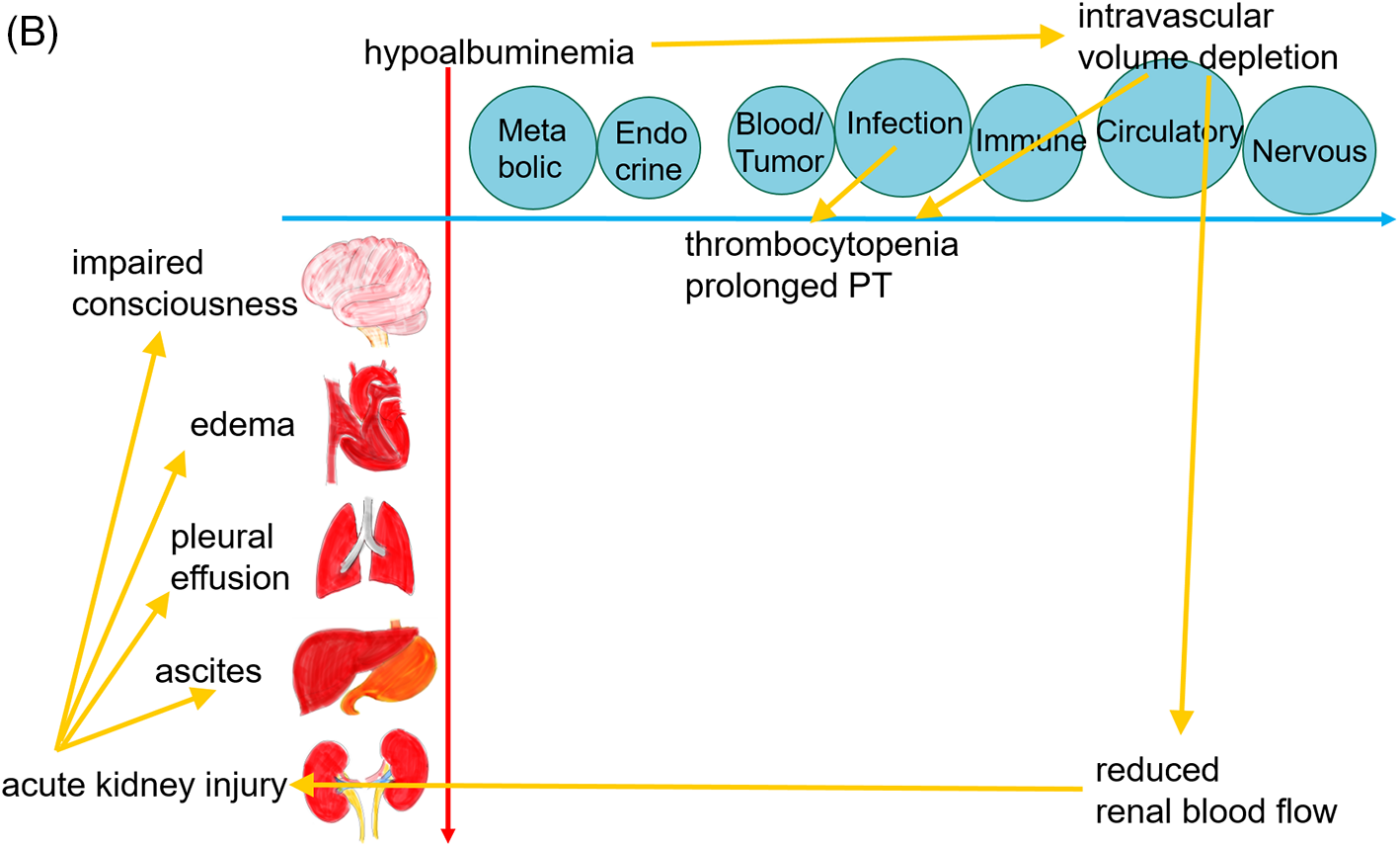

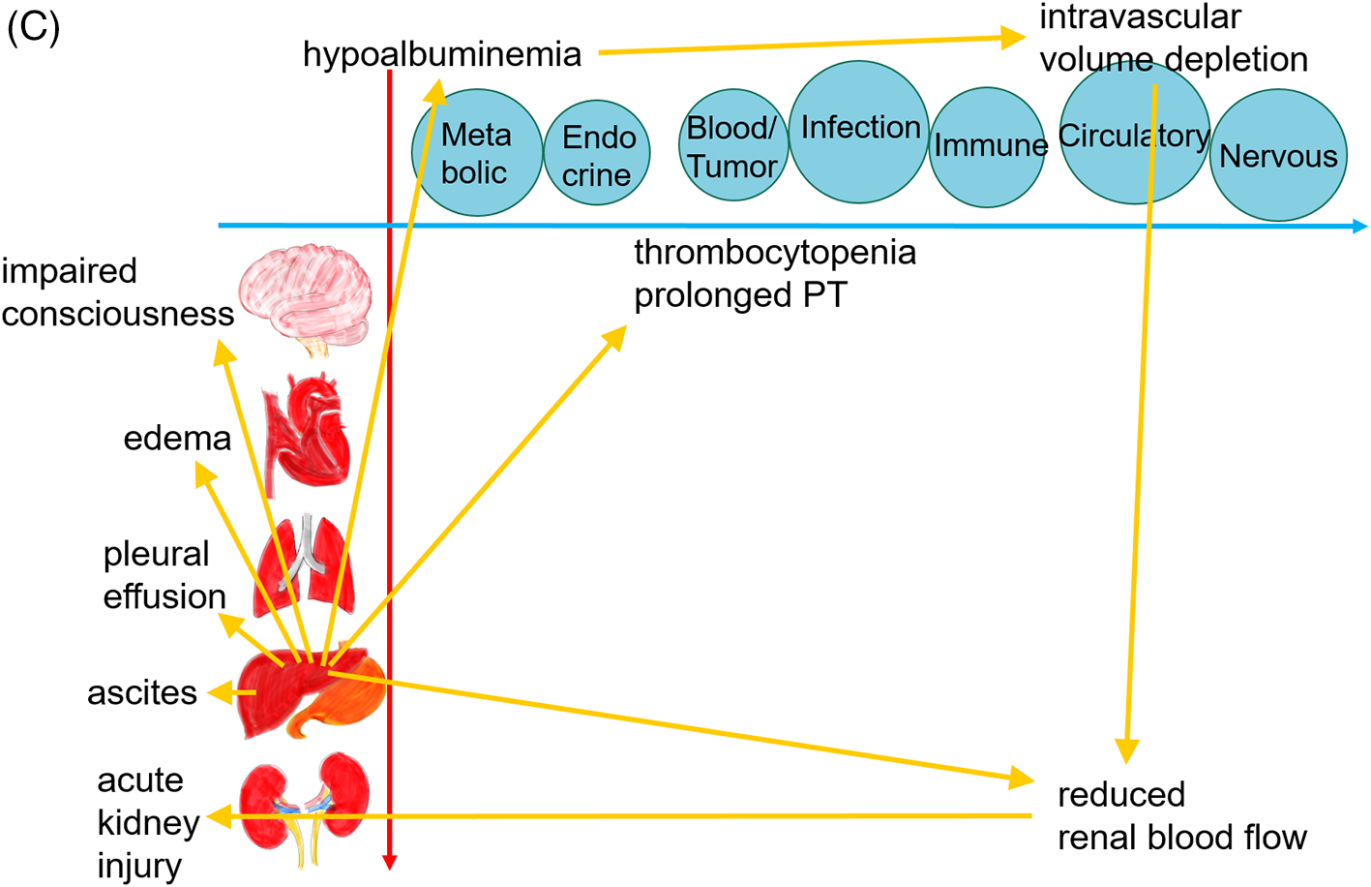
A 72-year-old woman presenting with AKI and edema.

A physician is consulted on a 72-year-old woman treated with antibiotics for osteomyelitis after an operation for a pressure ulcer. She has acute kidney injury (AKI) and edema. Physical examination shows altered consciousness level, pleural effusion, and ascites, while blood tests show hypoalbuminemia, thrombocytopenia and prolonged prothrombin time (PT). Urinalysis shows FeNa<1%, indicating reduced renal blood flow. The physician considers that hypoalbuminemia caused by malnutrition due to infection and surgery led to intravascular volume depletion, resulting in AKI and disseminated intravascular coagulation ( Figure 2A).

Arrow-head shows the relation between cause and effect. (A) Clinical reasoning map visualizing the pathophysiology considered by the physician. (B) Visualization of the pathophysiology considered by the physician using the 2D Approach. (C) The 2D Approach representing the underlying pathophysiology of the patient.

Various diagrammatic maps have been proposed for knowledge organization and clinical reasoning. Aconcept map ( Daley and Torre, 2010) and a cognitive map ( Wu *et al.*, 2014; Wu *et al.*, 2016) visualize patient’s presentation and the thinking process of physicians. In particular, a clinical reasoning map ( Jayasinghe, 2016) is useful for causal reasoning for complicated cases. Figure 2A is one example of these maps visualizing the causal reasoning for this patient.

When we convert this map to the 2D matrix, the pathophysiology would look like that demonstrated in Figure 2B. The pathophysiology starts from hypoalbuminemia caused by malnutrition due to infection and surgery according to the physician. Although hypoalbuminemia can be understood in the context of malnutrition, the 2D Approach provides a chance to look for other causes of hypoalbuminemia from the 2 perspectives of organs and systems. We can consider whether hypoalbuminemia (the metabolic system) might be a result of any organ dysfunction such as liver cirrhosis (liver/intestine) or nephrotic syndrome (kidney). We can also see that liver cirrhosis causes ascites, as well as thrombocytopenia and prolonged PT ( Figure 2C). This was the exact pathophysiology the patient had.

On the 2D matrix, we can map abnormal symptoms and signs on the most likely organs and systems, and visualize the problem list. Overviewing the problem list, we can link one symptom to another and visualize the pathophysiological hypothesis. In case we might inaccurately link symptoms and signs, the 2D Approach helps us notice on which point we mistook by visualizing the connection between abnormal symptoms and signs, and helps us reach an accurate pathophysiological understanding. In this sense, we can call the 2D matrix a “pathophysiology map”. Furthermore, the 2D Approach also helps us anticipate and prepare for further complications. In Figure 2C, physicians could anticipate that liver cirrhosis (cause) might be complicated by other manifestations of liver cirrhosis such as esophageal varices (effect), and thus be better prepared.

The difference between the 2D Approach and existing maps is that it visualizes the underlying pathophysiology of a patient based on the 2 view-points of organs and systems. These perspectives are essential in organizing knowledge.

### Visualization of clinical reasoning process

#### Case 2

**Figure 3.**
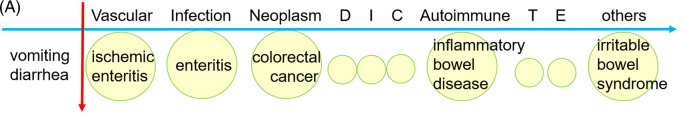

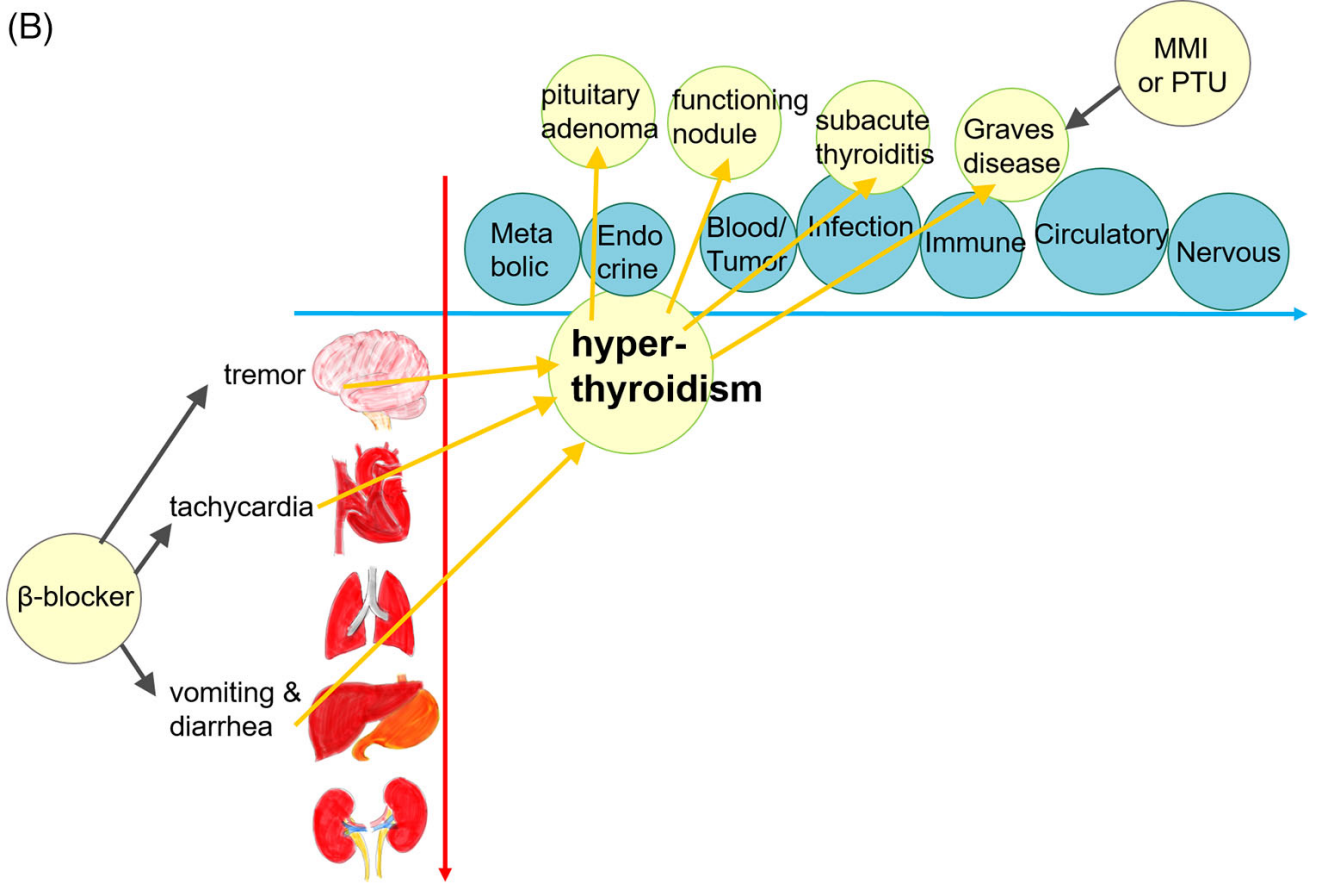
A 35-year-old man presenting with prolonged vomiting and diarrhea. (A) “VINDICATE” helps the physician make a list of differential diagnoses for vomiting and diarrhea. (B) The 2D Approach visualizes the clinical reasoning process from problem listing to disease management. Orange arrow-heads show the thinking process from symptoms to diagnosis, and gray arrow-heads show the thinking process from diagnosis to treatment.

A resident is consulted on a 35-year-old man presenting with vomiting and diarrhea for more than one month, where tachycardia and tremor were noted on physical examination. If the resident focuses on vomiting and diarrhea, his differential diagnoses would likely be enteritis, inflammatory bowel disease, colorectal cancer, ischemic enteritis, or irritable bowel syndrome, along with the broad categories included in VINDICATE (vascular, inflammation, neoplasm, degenerative, intoxication, congenital, autoimmune, trauma, endocrine and metabolic) ( Collins, 1981) ( Figure 3A), which is one of the well-known mnemonics developed informally for creating a differential diagnosis list, but has not been subjected to rigorous evaluation ( Ely, Graber and Croskerry, 2011).

However, this is not enough to understand the tachycardia and tremor. Using the 2D matrix, the resident needs to think about systems apart from organs to understand these systemic symptoms. He/she would probably notice that the endocrine system would cause these symptoms, especially hyperthyroidism ( Figure 3B). Then causes for hyperthyroidism will be diseases shown along with each system. This patient’s TSH receptor stimulating antibody was positive and a diagnosis of Graves’ disease was made. While Graves’ disease was treated with methimazole (MMI) or propylthiouracil (PTU), symptoms, such as tachycardia and tremor, were treated with β-blocker.

The 2D Approach may look like VINDICATE or a hybrid matrix table with etiologies and body systems ( Sacher and Detsky, 2009). The systems axis of the 2D Approach is similar to VINDICATE or etiologies on the hybrid matrix, as these represent the pathogenesis of diseases. However, the 2D Approach allows physicians to evaluate the likelihood of relevant diagnostic hypotheses after organizing case information based on the 2 view-points of organs and systems, whereas VINDICATE and the hybrid matrix simply help physicians recall and exhaustively evaluate the likelihood of diagnostic hypotheses. Furthermore, the 2D Approach visualizes the thinking process, not only of differential diagnosis but also of problem listing and patient management.

These 2 examples show how the 2D Approach visualizes underlying pathophysiology and the clinical reasoning process on a 2-dimensional matrix of organs and systems. We consider that the 2D Approach would help physicians organize their knowledge effectively and improve their clinical reasoning based on pathophysiological understanding. We also considered the difficulty learners may have because of inadequate knowledge in organizing their knowledge and improving their clinical reasoning skills ( Mamede *et al*., 2010).

We therefore tested the hypothesis that the 2D Approach helps learners with sufficient knowledge improve their clinical reasoning skills and that learners with inadequate knowledge will not improve their clinical reasoning skills even if they use the 2D Approach.

## Methods

This study was a prospective observational study. Participants were the 2017 postgraduate year 1 (PGY-1) residents (n=100) of the Tokyo Medical and Dental University Medical Hospital, corresponding to third-year medical students in the United States ( Nara, Suzuki and Tohda, 2011). All participants provided informed consent. They had a one-hour lecture on the 2D Approach, the contents of which were described in the introduction of this article. Before and after the lecture, the participants underwent written tests (pre- and post-tests). The lecture and 2 tests were a part of the residency program and took 2 hours to complete.

On the pretest, we assessed the participants’ baseline knowledge level (score for listing differential diagnoses for various symptoms), their baseline diagnosis accuracy (diagnostic accuracy for the case), and their baseline overall clinical reasoning ability (overall score). We used the score for listing differentials as the knowledge level score, because level of knowledge determines whether residents can list differentials for each symptom or not.

The pretest used Case 1, in which the patient presented with many symptoms as shown in the introduction, such as impaired consciousness level, edema, AKI, thrombocytopenia, prolonged PT, hypokalemia and metabolic alkalosis. The participants were asked to list differentials for each of these symptoms. For example, one question stated “List at least 4 differential diagnoses for edema.” to assess their knowledge about edema. They were also asked to choose the most likely diagnostic hypothesis for this case.

Using the pretest case as an example, they learned how to apply the 2D Approach to complicated cases during the lecture. The essence of the lecture was the 2 perspectives of organs and systems, and their cause-and-effect relationship, which make visible the pathophysiology of a patient and the clinical reasoning process of a physician on the 2D matrix of organs and systems.

The posttest used a different case from the pretest, but consisted of the same types of questions as the pretest. The case presented with a diversity of problems, which included impaired consciousness level, shock, renal dysfunction, hypoglycemia, hyponatremia, hyperkalemia, and metabolic acidosis. The participants were asked to list differentials for each symptom and to choose the most likely diagnostic hypothesis for the case. During the posttest, the participants were allowed to take notes on the 2D matrix. In case participants left notes on the 2D matrix, we determined that the participants visualized their clinical reasoning process using the 2D Approach and defined them as the 2D Approach users, with the intension to compare the change in the pre- and post-test scores between the 2D Approach users and non-users.

**Figure 4.**
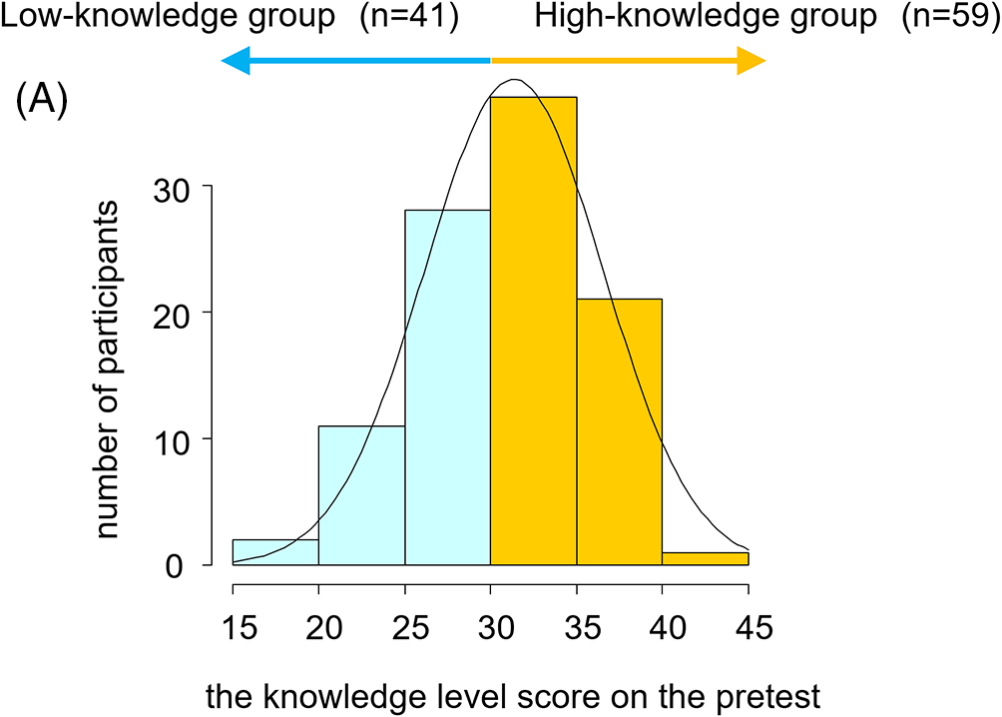

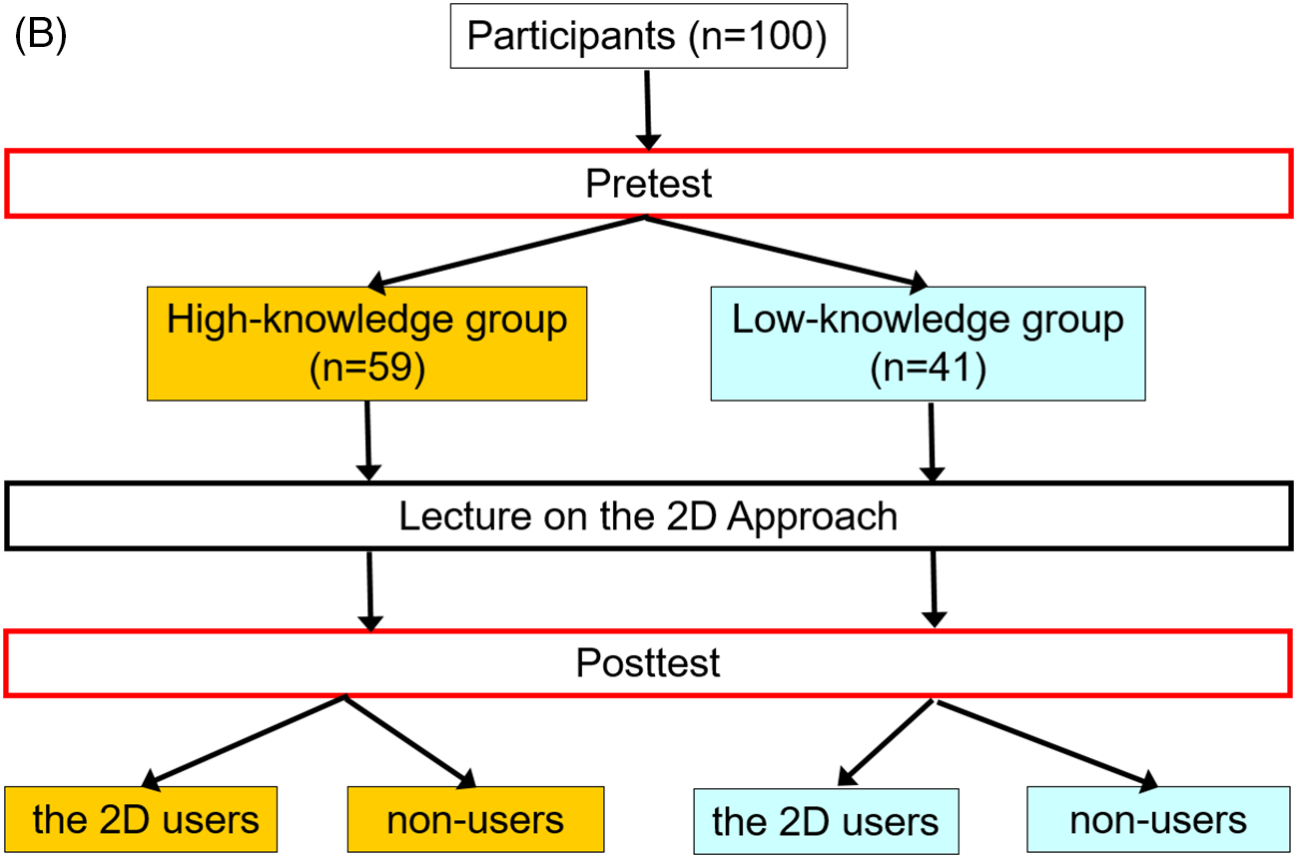
(A) The pre-existing knowledge level scores of the participants. (B) Study design.

As we hypothesized that the pre-existing knowledge level is a confounding factor between the 2D Approach use and the improvement in clinical reasoning skills, we stratified the participants according to their baseline knowledge level score on the pretest, into 2 groups (the high-knowledge group and the low-knowledge group). The participants (n=59) who got scores no less than 31 were categorized into the high-knowledge group, while the participants (n=41) who got scores less than 31 into the low-knowledge group. The cut-off point in the knowledge level score was automatically determined by the statistical software ‘EZR’ (Easy R) version 1.34 ( Figure 4A). In each of the 2 knowledge level groups (high-knowledge and low-knowledge), we compared the change in the pre- and post-test scores between the 2D Approach users and non-users by repeated-measures analysis of variance (ANOVA) ( Figure 4B).

We developed test cases based on real-world patients for which physicians need a wide range of knowledge to make a diagnosis. Based on the grading criteria developed *a priori* on the consensus of the authors (the perfect score for both the pre- and post-tests were 51 points), we pilot tested them on the 2016 PGY-1 residents of the hospital (n=108). Cronbach alpha reliability was 0.64 for the pretest and 0.64 for the posttest.

All analyses were performed with EZR (Saitama Medical Center, Jichi Medical University, Saitama, Japan), which is a graphical user interface for R (The R Foundation for Statistical Computing, Vienna, Austria). More precisely, it is a modified version of R commander designed to add statistical functions frequently used in biostatistics ( Kanda, 2013). This study was approved by the Institutional Review Board of Tokyo Medical and Dental University.

## Results/Analysis

Among all the participants, 56 participants out of 100 used the 2D Approach during the posttest. In the high-knowledge group (n=59), 33 participants used the 2D Approach and the remaining 26 participants did not. In the low-knowledge group (n=41), 23 participants used the 2D Approach on the posttest and 18 participants did not. In both groups, the high-knowledge group and the low-knowledge group, there was no statistically significant difference in the baseline scores on the pretest (the knowledge level score, the diagnostic accuracy, the overall score) between the 2D Approach users and the non-users ( Table 1).

**Table 1.** Comparison of the baseline scores on the pretest between the 2D Approach users and non-users in each of the 2 knowledge level groups (high-knowledge and low-knowledge).

	High-knowledge group (n=59)			Low-knowledge group (n=41)		
	2D users (n=33)	Non-users (n=26)	P value	2D users (n=23)	Non-users (n=18)	P value
Knowledge level score, mean (SD)	34.52 (3.12)	35.35 (2.76)	0.29	26.57 (2.98)	25.94 (3.26)	0.53
Correct diagnosis, n (%)	11 (33)	7 (27)	0.78	2 (8.7)	5 (28)	0.21
Overall score, mean (SD)	37.39 (4.58)	38.15 (4.58)	0.53	27.65 (3.98)	27.72 (3.46)	0.95

**Figure 5.**
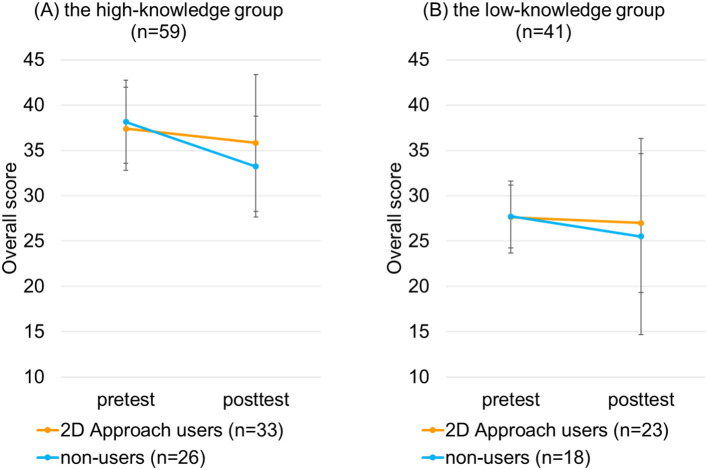
Comparison of the change in the pre- and post-test scores between the 2D Approach users and non-users by repeated measures ANOVA, in each of the 2 knowledge level groups (high-knowledge and low-knowledge).

In the high-knowledge group, the change in the pre- and post-test overall scores was significantly larger among the 2D Approach users than non-users (-1.58 (95%CI: -4.09~0.94) vs -4.92 (95%CI: -6.9 ~ -2.93), p=0.046, repeated measures ANOVA) ( Figure 5A). In contrast, in the low-knowledge group, the change of the scores was not significantly different between the 2D Approach users and non-users (-0.65 (95%CI: -3.99~ 2.68) vs -2.22 (95%CI: -7.61 ~ -3.17), p=0.59, repeated measures ANOVA) ( Figure 5B).

## Discussion

Whether residents use the 2D Approach or not was not associated with the baseline knowledge level or the other scores (diagnostic accuracy, overall score) on the pretest ( Table 1). It seems that it depends on each resident whether they use the 2D Approach or not.

We compared the change of the clinical reasoning skills between the residents who used the 2D Approach during the posttest and those who did not, in each of the 2 knowledge level groups. As we had expected, their knowledge level influenced the results. In the high-knowledge group, the 2D Approach users improved their scores more than the non-users. On the other hand, in the low-knowledge group, use of the 2D Approach was not associated with improvement in the scores. These findings imply that residents need sufficient knowledge to use the 2D Approach effectively in improving their clinical reasoning skills.

Norman *et al*. (2017) point out that knowledge deficits are a significant contributor to diagnostic error, and strategies to induce some reorganization of knowledge appear to have small but consistent benefits. In this context, we can say that the 2D Approach is not a method to help users acquire knowledge but a framework to help users reorganize their previously acquired knowledge for clinical reasoning.

They also add that the level of the learners and the difficulty of the cases affect its impact, that is, analytical thinking based on the retrieval and reorganization of diagnostically relevant knowledge results in improvement in solving simple cases for novices, while for more advanced clinicians it is effective for solving complex cases ( Mamede *et al*., 2010).

In our study, we selected the PGY-1 residents in Japan as participants, who are corresponding to third-year medical students in the United States ( Nara, Suzuki and Tohda, 2011). In Japan, undergraduate students have little chance to diagnose complicated cases, while most PGY-1 residents face difficulty in diagnosing and managing complicated cases and they are eager to learn a new method for clinical reasoning. We also considered that Case 1 and 2 may be difficult for undergraduate students, and carefully selected the PGY-1 residents as participants. Besides, we stratified the PGY-1 residents according to their baseline knowledge level on the pretest, and detected the effect of the 2D Approach on clinical reasoning only among the residents with sufficient knowledge.

We used complicated cases as the pre- and post-tests to assess their improvement in clinical reasoning. There remains a possibility that the 2D Approach can help residents without sufficient knowledge diagnose simple cases. This might indicate case specificity. However, we used representative cases presenting with diverse symptoms and signs which cover various organs and systems, and asked residents to list differentials for each symptom and to choose the most likely diagnosis for the case. We consider that we were able to assess a wide range of knowledge and clinical reasoning skills. Further study is needed to investigate the effect of the 2D Approach on different grades of students or residents, and also to assess their ability by using various levels of cases, from simple to complicated ones.

There are some more limitations in this study. All the residents attended the same lecture on the 2D Approach as it was a part of the residency program in the hospital, and the comparison was between the residents who left notes on the 2D matrix and those who did not. We might have missed some participants who used the 2D Approach in their mind but did not leave notes. However, it would be difficult for residents to use the 2D Approach in their mind just after learning this method, and we consider that it is more important to make the reasoning process visible on the 2D matrix than to use it in mind in order to organize their knowledge and improve their clinical reasoning skills. Lastly, generalization of the findings of this study is limited due to the single location of our research.

## Conclusion

On condition that residents have sufficient knowledge, the 2D Approach helps them organize their knowledge and improve their clinical reasoning skills, through visualization of pathophysiology on a matrix of organs and systems.

## Take Home Messages

• The 2D Approach with a matrix of organs and systems visualizes pathophysiology.
• The 2D Approach visualizes the clinical reasoning process from problem listing to patient management.
• By using the 2D Approach, more precisely, by visualizing the reasoning process on the matrix of organs and systems, residents with sufficient knowledge can organize their knowledge and improve their clinical reasoning skills.

## Notes On Contributors

Dr. Hisashi Shimozono is a PhD student, Department of Medical Education Research and Development, Graduate School of Medical and Dental Sciences, Tokyo Medical and Dental University, Japan. (ORCID number: https://orcid.org/0000-0001-7670-5486)

Dr. Makoto Takahashi is an Associate Professor, Department of Medical Education Research and Development, Graduate School of Medical and Dental Sciences, Tokyo Medical and Dental University, Japan. (ORCID number: https://orcid.org/0000-0001- 5810-1224)

Mr. Makoto Tomita is an Associate Professor, Clinical Research Center, Tokyo Medical and Dental University Hospital of Medicine, Japan.

Dr. Kazuki Takada is a Professor, Department of Professional Development in Health Sciences, Graduate School of Medical and Dental Sciences, Tokyo Medical and Dental University, Japan.

Dr. Yujiro Tanaka is a Professor, Department of Medical Education Research and Development, Graduate School of Medical and Dental Sciences, Tokyo Medical and Dental University, Japan. (ORCID number: https://orcid.org/0000-0002-3895-1886)

## Declarations

The author has declared that there are no conflicts of interest.

## Ethics Statement

This study was approved by the Institutional Review Board of Tokyo Medical and Dental University (M2016-132).

## External Funding

This article has not had any External Funding
