# Supplementary material for: “The 2-Dimensional Approach”: a novel tool to help learners organize their knowledge and improve their clinical reasoning skills
Source: MedEdPublish (2016). 2019 Jun 17;8:134. [Version 1] doi: 10.15694/mep.2019.000134.1 (PMC10712542; doi:10.15694/mep.2019.000134.1)
Supplement: Peer review history

# Reviewer report: Reviewer response for version 1

**DOI:** 10.21956/mep.19845.r29934

**Published:** 2019-09-01

**Authors:** Trevor Gibbs

**Affiliations:** 1 AMEE

This review has been migrated. The reviewer awarded 4 stars out of 5 For students to understand clinical reasoning they need to know how the basic sciences and the clinical sciences integrate to provide the true clinical diagnosis. This paper explores an interesting approach to bring the two sciences together. The paper describes a somewhat complicated approach which appeared of value to the student. I would agree with my fellow reviewer that the paper needs a couple of read-throughs to understand before it's novelty comes to the fore. I did wonder whether the cases were true or constructed, based upon learning outcomes and whether the outcomes themselves were long-lasting ( as would be seen if the evaluation was constructed over a longer period of time. Moving up the Kirkpatrick levels would be better here) I enjoyed however the novelty, the very good reference list and the bravery of the authors in submitting this paper and I do believe that it is a paper worthwhile reading by all involved in curriculum planning.

Reviewer Expertise:

NA

No decision status is available

# Reviewer report: Reviewer response for version 1

**DOI:** 10.21956/mep.19845.r29935

**Published:** 2019-06-30

**Authors:** Julie Hunt

**Affiliations:** 1 Lincoln Memorial University

This review has been migrated. The reviewer awarded 4 stars out of 5 The authors describe a framework for visualizing or organizing knowledge to improve clinical reasoning in complicated medical cases. They report the use of this framework by a cohort of medical students (first year Japanese residents), among whom the higher-knowledge students perform better on clinical reasoning on post-test. The 2D approach expands upon the currently taught mnemonics (e.g. VINDICATE) to encourage the user to consider the interaction of organs and systems to reach a more accurate understanding of the pathophysiology of disease.This paper describes the tool well, although it might have been helpful to have an expert's step-by-step explanation of both cases and how (in what order) the cases were framed using the 2D approach. I would like to see additional work done with this tool, including what the impact was on clinical reasoning after a longer training intervention than a 1-hour lecture, perhaps after workshop training and practice use, and after a longer retention time. Based on the initial results I think this tool shows potential for assisting students with clinical reasoning better than the currently taught mnemonics.

Reviewer Expertise:

NA

No decision status is available

# Reviewer report: Reviewer response for version 1

**DOI:** 10.21956/mep.19845.r29936

**Published:** 2019-06-18

**Authors:** Johnny Lyon Maris

**Affiliations:** 1 Southampton GP Education Unit

This review has been migrated. The reviewer awarded 3 stars out of 5 This is a well referenced article and trying to conceptualise clinical reasoning is an admirable object, thankyou for submitting. However I had to read and re-read this article a number of times to understand the concept and I still do not think I have understood it. I can see that a diagnosis can use the grid to see the potential complications of the clinical course but I am finding it hard to see how the symptoms can lead to a differential using this approach, or have I misunderstood it completely?In addition I was unsure whether the differing sizes of the blue circles along the horizontal had any relevance?

Reviewer Expertise:

NA

No decision status is available
